# The relation between immunologic variables and symptom factors in patients with major depressive disorder

**DOI:** 10.1186/s12991-018-0201-7

**Published:** 2018-07-26

**Authors:** Yang-Whan Jeon, Sang-Ick Han, E. Jin Park

**Affiliations:** Department of Psychiatry, College of Medicine, Incheon St. Mary’s Hospital, The Catholic University of Korea, 56 Dongsu-ro, Bupyeong-gu, Incheon, 21431 South Korea

**Keywords:** Major depressive disorder, Immunologic variables, Anxiety, Selective serotonin reuptake inhibitor

## Abstract

**Background:**

The associations between depression and immunity were investigated by measuring the scores of Hamilton Rating Scale for Depression (HRSD) and peripheral lymphocyte parameters in patients with major depressive disorder (MDD).

**Methods:**

Forty-nine patients with MDD were recruited and their clinical symptoms are evaluated with 17-item HRSD which was factorized using the confirmatory factor analysis (i.e., depression factor, insomnia factor, and anxiety factor). Basic immunologic variables such as CD4, CD8, and CD56-positive cell numbers were measured by flow cytometry. Natural killer cell activity (NKCA) was also assessed by ELISA method using K-562 cells as target cells. All patients were treated for 4 weeks with selective serotonin reuptake inhibitors. Immunologic and clinical variables were measured both at baseline and after medication.

**Results:**

CD8-positive cell number was increased (*p* < .05) and CD4/CD8 ratio was decreased (*p *< .01) after medication. NKCA showed a significant positive correlation with anxiety factor scores of HRSD (*p *< .05) at baseline. However, except NKCA, there was no correlation between other immunologic measures and symptom factors.

**Conclusion:**

These results suggest that immunologic measure such as NKCA may be an important variable for symptom of MDD such as anxiety during acute depressive state.

## Introduction

Major depressive disorder (MDD) is one of the most common mental disorders. 10–25% of women and 5–10% of men may become susceptible to the condition once or more in their life [1]. Patients with MDD have a mortality rate over twice higher than normal and MDD is also known as a risk factor that can increase the morbidity of various medical diseases [2, 3]. Many researchers have demonstrated that changes of immune functions may play an important role in increasing mortality and morbidity among patients with MDD [4, 5].

However, there were controversial reports on immune functions in patients with MDD: immunosuppression or activation of the immune system [6, 7]. Several methods had been used to measure lymphocytes, determine how the lymphocytes function, and assess markers of immune activity in patients with MDD. There were different reports on the number of lymphocytes: the number of lymphocytes or natural killer (NK) cells has increased, decreased, or unchanged [8–12]. The functions of lymphocytes have been assessed on the basis of lymphocyte proliferation by mitogen stimulation, NK cell activity (NKCA), and the cytokine secretion. Many researchers have reported that lymphocyte proliferation by mitogen was decreased and a consistent decrease in NKCA in patients with MDD [9, 13]. However, no consistent report has been made on the number of lymphocytes in patients with MDD and limited research has been conducted on their immune functions in Korea.

These controversial results are probably due to interrelationship with the immune system and heterogeneity among patients with MDD. The patients with MDD diagnosed according to the criteria in the fourth edition of the diagnostic and statistical manual of mental disorders (DSM-IV) are not identical to one another but show quite different clinical symptoms [14]. For this reason, many researchers have tried to categorize MDD according to the aspect of melancholy or the subtype of accompanying symptoms in examining the immune system [15, 16]. It is, therefore, necessary to classify a diversity of clinical symptoms of MDD in valid way. This study employed the 17-item Hamilton Rating Scale for Depression (HRSD), which has been widely used in clinical practice and research, to examine immune functions in patients with MDD in terms of those symptom factors divided by confirmational factor analysis [17]. Many studies have examined the factor structure of the HDRS, with the original 17-item version of the scale being the most studied. Though some evidence of a relatively stable factor has been provided, not all studies of the 17-item scale factor support this finding [17–23].

As the literature review found that fluoxetine, a selective serotonin reuptake inhibitor (SSRI), was effective in restoring T cells negatively affected by stress and that NK cells became more active as depressive symptoms improved, an attempt was made to investigate immunologic variables before and after medication in patients with MDD [24–26].

This study aimed to examine immunologic changes by SSRI in patients with MDD and determine correlation between the symptom factors in HRSD and the immunologic variables. We hypothesized that immunologic variables would change after medication and some symptom factors would be correlated with immunologic variables.

## Methods

### Subjects and symptom assessment

This study was conducted in 49 outpatients diagnosed with MDD at the department of psychiatry, Incheon St. Mary’s Hospital in the Catholic University of Korea. Diagnosis of MDD was confirmed by structured clinical interview for DSM-IV by two psychiatrists (EJP and SIH) [27]. The subjects were treated with SSRIs and doses of SSRIs varied depending on patients’ symptom improvement and side effects. Those who were at high risk of committing suicide or had psychotic symptoms, who had history of bipolar disorder, drug or alcohol dependence, schizophrenia, or other psychotic disorders, and who had a history of taking medication on a current episode of MDD within a month were excluded. Patients with abnormal findings in a battery of clinical laboratory test (including urine analysis, complete blood count, renal and liver function test, mineral panel, thyroid indices, chest X-ray, and electrocardiogram) were excluded. Those who had suffered from an immunologic disease or a malignant tumor were also excluded. In addition, those who had taken any other medicine during the 4 week period of treatment or who were found to have suffered from a cold or symptoms of inflammation by medical history or physical examinations were excluded. 17-item HRSD was used to assess the symptoms of MDD in the subjects at baseline and after 4 week treatment. HRSD was applied by trained psychiatric residents. EJP had trained the residents for interrater reliability using video recordings of ten cases of patients with MDD. This study was conducted with the approval of the Institutional Review Board of Incheon St. Mary’s Hospital, the Catholic University of Korea (IRB number OCMC07MI002) and all the participants were given a full explanation of its purpose before they made a written consent. The participants signed the consent form accepting to participate and also for permission to published.

### Flow cytometry

A peripheral blood sample was taken from each subject at baseline and after 4 week treatment. The peripheral blood was treated with EDTA for flow cytometric analysis. All the tests were performed within 4 h after sampling and each sample was stored at room temperature (18–20 °C) before the analysis. To examine the immune status of the subjects, CD4-positive (helper T cell), CD8-positive (suppressor T cell), and CD56-positive cells (NK cell) were counted in the following way: 50 µL of the whole blood sample treated with EDTA was mixed with 5 µL of an antibody to each antigen (PE-CY5 conjugated mouse anti-human CD3, FITC conjugated mouse anti-human CD4, PE conjugated mouse anti-human CD8, and PE conjugated mouse anti-human CD56) (Immunotech, Marseille, France); then, each mixture was incubated in a darkroom for 30 min. Erythrocytes were then lysed by incubation in 2 mL of lysing solution (Immunotech, Marseille, France) and the sediments were washed in phosphate-buffered saline. Fluorescence was analyzed by flow cytometry (FACScan, Becton–Dickinson, CA, USA). Staining of CD3, CD4, and CD8 was performed by triple stain, and each positive population of CD4 and CD8 was separated from the gating cells with CD3-positive lymphocytes. CD56 were stained with a single preparation. Isotype controls (Immunotech, Marseille, France) were stained as the negative control, and the percentage of positive signal group in the gated lymphocytes was calculated.

### Assessment of natural killer cell activity

To determine the cytotoxic activity of NK cells on the K-562 tumor cell line (ATCC, Rockville, MD, USA), we used a modified lactate dehydrogenase (LDH) release assay. Mononuclear cells as effector cells, at a concentration of 8 × 10^5^/100 mL in a 200 µL culture medium, were mixed with K-562 cells, as target cells, at a concentration of 1 × 10^4^/100 mL. The assay was performed in 96-well U-bottom culture plates (Corning Glass Works, Corning, NY, USA) and incubated for 4 h at 37 °C in a 5% CO_2_ humid atmosphere. After the reaction, we sampled a 100 µL medium and used ELISA (Roche Diagnostics, Roche, Mannheim, Germany) to measure the amount of LDH isolated from K-562 cells as follows: we transferred the medium to a flat-bottom microplate (Molecular Devices Co., Sunnyvale, CA, USA) and added the same dose of a reagent (LDH substrate mixture) to it, and let it react at room temperature (15–25 °C) with light blocked out for 30 min, and measured absorbance at 490 nm wavelength. Culture media was used for the spontaneous LDH assay, and Triton X-100 solution was added to the media to determine the maximum amount of LDH isolated from K-562 cells, so that the cells could be lysed completely. On the basis of the absorbance in each condition, the following equation was used to assess activity of NK cells (% cytotoxicity):

$${\text{Natural killer cell activity}}\left( {{\% }\;{\text{cytotoxicity}}} \right) = \frac{{{\text{LD}}{{\text{H}}_{\text{experimental}}} - {\text{LD}}{{\text{H}}_{\text{effector cells}}} - {\text{LD}}{{\text{H}}_{\text{spontaneous}}}}}{{{\text{LD}}{{\text{H}}_{\text{maxiaml}}} - {\text{LD}}{{\text{H}}_{\text{spontaneous}}}}} \times 100$$

LDH_experimental_ is the value obtained by culturing both NK cells and K-562 cells, LDH_effector cells_ is the value obtained by culturing NK cells separately, LDH_spontaneous_ is the value obtained by culturing K-562 cells separately, and LDH_maximal_ is the value obtained from K-562 cells cultured with triton X-100 added.

### Statistical analyses

The factor analysis divided the 17-item HRSD into depression factors (Fd), insomnia factors (Fi), and anxiety factors (Fa) by confirmational factor analysis. Fd included item 1 (depressed mood), item 2 (feeling of guilt), item 7 (work and activities), item 8 (retardation: psychomotor), and item 13 (somatic symptoms general); Fi included item 4 (insomnia early), item 5 (insomnia middle), and item 6 (insomnia late); and Fa included item 9 (agitation), item 10 (anxiety: psychological), item 11 (anxiety: somatic), item 15 (hypochondriasis), and item 16 (loss of weight). *T* test and one-way analysis of variances were used to compare the immunologic variables in terms of the demographic data and the clinical characteristics. Bonferroni test was applied for age group. To determine if immunologic variables differed across age and whether other demographic data impacted immunologic variables, we conducted a series of multivariable linear regression analysis. In these models, we included age and recent episode. Paired *T* test was used to compare the total scores of HRSD, the scores for three symptom factors, the number of lymphocyte subtypes, and the number and activity of NK cells at baseline and after medication. To determine the correlation between the number of lymphocyte subtypes, the number and activity of NK cells, and the three symptom factors of HRSD, we used Pearson’s method and compared *r* and *p* values at baseline and after treatment after controlling age and recent episode. The significance level for all the statistics was set at .05. Statistical processing was performed using Statistica (version 12.0)

## Results

### Demographics and clinical characteristics

Forty-nine patients with MDD were included. Table 1 presents the demographic and clinical characteristics. Subjects were treated with escitalopram (*N* = 27; mean daily dose, 12.8 mg; range, 5–20 mg/day), paroxetine (*N* = 12; mean daily dose, 50 mg; range, 25–50 mg/day), and sertraline (*N* = 10; mean daily dose, 75 mg; range, 50–100 mg/day). Response to treatment was defined as ≥ 50% reduction of the baseline HAM-D score after 4 week treatment.

**Table 1:** Demographic and clinical characteristics of the subjects at baseline

Variables	Number (%)
Age (years)	50.9 ± 13.6
20–39	11 (22.4)
40–59	24 (49.0)
60≦	14 (28.6)
Sex	
Males	7 (14.3)
Females	42 (85.7)
Onset age (years)	48.1 ± 15.5
Recent episode	
Single	26 (53.1)
Recurrent	23 (46.9)
Duration of current episode (months)	2.0 ± 2.2
Response	
Responders	23 (46.9)
Non-responders	26 (53.1)

### Immunologic variable and HRSD scores

For the number of lymphocyte subtypes and the number and activity of NK cells by the demographic and clinical characteristics of the subjects at baseline, there were significant differences in CD4/CD8 ratio (*p *= .04) and NKCA (*p *= .01) among age groups. In addition, single episode group had significantly greater NKCA (*p *= .01) (Table 2).

**Table 2:** Descriptive statistics of lymphocyte subsets and NKCA at baseline by the characteristics of the subjects

Variables	CD3, %	CD4, %	CD8, %	CD4/CD8, %	CD56, %	NKCA, %
Age (years)						
20–39	66.0 ± 9.0	38.7 ± 6.1	27.2 ± 4.3	1.4 ± .2	16.8 ± 6.7	9.8 ± 7.6
40–59	64.1 ± 8.4	40.8 ± 6.1	23.3 ± 5.7	1.9 ± .6*	18.4 ± 6.6	17.1 ± 11.1
60≦	61.1 ± 9.9	39.3 ± 5.6	21.8 ± 6.1	1.9 ± .5	21.2 ± 8.8	22.6 ± 11.2^†^
Sex						
Males	63.1 ± 11.3	40.0 ± 7.9	23.2 ± 6.3	1.8 ± .5	21.2 ± 9.3	17.1 ± 7.6
Females	63.7 ± 8.7	39.9 ± 5.7	23.8 ± 6.0	1.8 ± .5	18.4 ± 7.0	17.0 ± 11.8
Recent episode						
Single	62.4 ± 8.3	38.9 ± 6.1	23.4 ± 5.6	1.8 ± .5	20.5 ± 8.1	20.3 ± 12.3
Recurrent	65.1 ± 9.7	41.0 ± 5.7	24.1 ± 6.1	1.8 ± .5	17.0 ± 6.1	13.4 ± 8.8*
Response						
Responders	62.8 ± 8.7	40.0 ± 5.5	22.8 ± 5.2	1.8 ± .5	20.8 ± 7.9	17.0 ± 9.7
Non-responders	64.4 ± 9.4	40.0 ± 6.4	24.6 ± 6.2	1.7 ± .6	17.1 ± 6.5	17.1 ± 12.6

* *p* < .05, a significant difference between 20 and 39 years and 40–59 years by Bonferroni test

^†^ *p *< .05,a significant difference between 20 and 39 years and 60≦ years by Bonferroni test

**Fig. 1:**
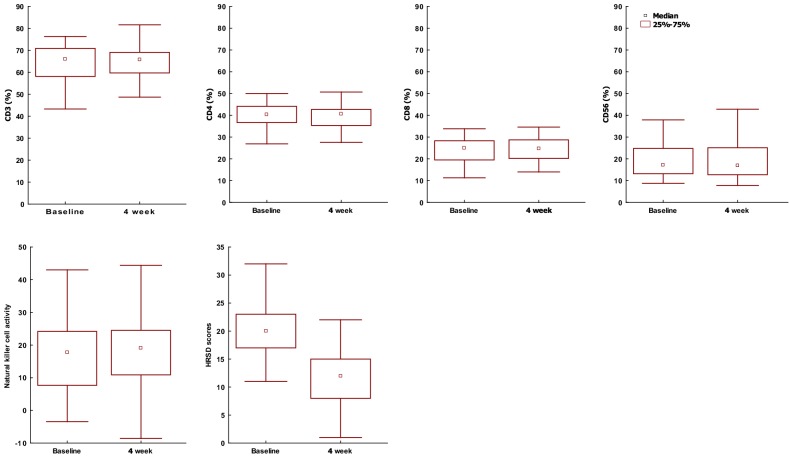
Plots of immunologic variables and Hamilton Rating Scale for depression scores at baseline and after 4 week treatment

Figure 1 presents plots of immunologic variables and HRSD scores at baseline and after 4 week treatment. After controlling for age and recent episode, there were significant increase CD8-positive cells (*p *= .03) and decrease CD4/CD8 ratio (*p *= .02) after medication. After 4 week treatment, both total score of HRSD and the scores of Fd, Fa, and Fi were significantly lower (Table 3).

**Table 3:** Immunologic variables and symptom factor scores in patients with major depressive disorder at baseline and after 4 week treatment

Variables	At baseline	After treatment	*t* value	*p*
CD3 (%)	63.6 ± 9.0	64.7 ± 8.9	− 1.1	N.S.
CD4 (%)	39.9 ± 5.9	39.9 ± 6.0	.0	N.S.
CD8 (%)	23.8 ± 5.8	24.8 ± 5.4	− 2.2	.03.
CD4/CD8	1.8 ± .5	1.7 ± .4	3.3	.002.
CD56 (%)	18.8 ± 7.3	19.6 ± 8.7	− 1.1	N.S.
NKCA (%)	17.0 ± 11.2	18.5 ± 11.6	− 1.0	N.S.
HRSD, total	20.3 ± 5.5	11.4 ± 5.2	9.1	< .001
Fd	7.3 ± 2.3	4.3 ± 2.3	8.4	< .001
Fi	3.4 ± 1.5	1.3 ± 1.5	7.8	< .001
Fa	4.1 ± 1.7	2.5 ± 1.4	6.0	< .001

### Partial correlation between immunologic variables and HRSD scores

At baseline, NKCA was positively correlated with Fa (*r *= .35, *p *= .02). After treatment, Fi was positively correlated with CD4/CD8 (*r *= .33, *p *= .03) (Table 4).

**Table 4:** Partial correlation between immunologic variables and symptom factor scores in patients with major depressive disorder before and 4 weeks after the treatment after controlling age and recent episode

Variables	Before treatment			After treatment				
	HRSD (total)	Fd	Fi	Fa	HRSD (total)	Fd	Fi	Fa
CD3	*r* = .11	*r* = .13	*r* = .10	*r* = .07	*r* = .11	*r* = .13	*r* = − .01	*r* = .14
	*p *= .47	*p *= .40	*p* = .51	*p* = .65	*p* = .79	*p* = .39	*p* = .97	*p* = .36
CD4	*r* = .02	*r* = − .02	*r* = .09	*r* = − .03	*r* = .13	*r* = .04	*r* = .17	*r* = .11
	*p* = .87	*p* = .88	*p* = .54	*p* = .82	*p* = .84	*p* = .80	*p* = .25	*P* = .48
CD8	*r* = .15	*r* = .23	*r* = .06	*r* = .25	*r* = .03	*r* = .16	*r* = − .21	*r* = .10
	*p *= .32	*p* = .11	*p *= .69	*p* = .32	*p* = .84	*p* = .27	*p* = .16	*p* = .51
CD4/CD8	*r* = − .14	*r* = − .21	*r* = − .03	*r* = − .13	*r* = .03	*r* = − .18	*r* = .33	*r* = − .02
	*p *= .37	*p* = .15	*p* = .85	*p* = .37	*p* = .82	*p* = .23	*p* = .03*	*p* = .87
CD56	*r* = − .03	*r* = .00	*r* = .03	*r* = − .08	*r* = − .13	*r* = − .01	*r* = − .08	*r* = − .25
	*p* = .84	*p* = .99	*p* = .84	*p* = .61	*p* = .39	*p* = .94	*p* = .58	*p* = .09
NKCA	*r* = .13	*r* = .23	*r* = − .23	*r* = .35	*r* = − .05	*r* = .00	*r* = − .09	*r* = − .01
	*p* = .38	*p* = .12	*p* = .12	*p* = .02*	*p* = .76	*p* = .98	*p* = .53	*p* = .97

* *p *< .05

## Discussion

The patients with MDD in this study were significantly increased in the number of CD8-positive cells and, consequently, a significant decrease in the CD4/CD8 ratio after medication. Severe depression was associated with lower CD8-positive cells in a large-scale meta-analysis among medically healthy persons, and as severity of depression may decrease after medication, CD4/CD8 ratio is correlated with the severity of depression [9, 28].

Medication made no change in NKCA in this study. Frank et al. reported that the responders to medication shown increased NKCA after the medication, whereas the non-responders shown decreased; others found that the decrease in NKCA lasted for 6 months during which medication was given [26]. No change in NKCA by medication in this study is inconsistent with the previous findings probably because of the differences in subjects’ demographic variables. In this study, 47% of subjects responded and 53% made no response after 4 week treatment. The ratio of responders seems to be relatively low, given that about 67% of patients with MDD are usually expected to respond to medication. Further research needs to execute long-term follow-up of NKCA in patients with MDD, since some non-responders are likely to respond to the medication 6–8 weeks later [29].

Our model contained three symptom factors of HRSD: Fd, Fi, and Fa. Correlation between these factors and the immunologic variables was assessed at baseline and after treatment. NKCA was significantly positively correlated with anxiety factors in HRSD at baseline. More than half of individuals with a lifetime history of MDD report a lifetime history of one or more anxiety disorder, with the anxiety disorders predating the onset of MDD in the majority of cases and that depression disorder is accompanied by anxiety symptoms in most cases and immunologic research concerning anxiety has found that depressed patients with panic disorder may have a larger number of T cells and mitogen stimulation make greater lymphocyte proliferation than those without panic disorder and NKCA positively correlated with the score for Taylor’s manifest anxiety in cancer patients [30–35]. From these findings, the result that symptoms of anxiety were correlated with the immunologic variables in patients with MDD may provide a clue to the immunologic changes observed among patients with MDD.

We analyzed by controlling two demographic variables, which showed difference in immunologic measures at baseline and after medication. (1) Age. The age group from 40 to 59 years accounted for 49% and older patients had significantly greater NKCA. The early reports suggested that NK cell numbers and activity were unchanged with aging, but more recent investigators have generally described an increase in the proportion of CD56^dim^ (mature) NK cells, a decrease in the number, and/or activity of NK cells, with a decreased affinity for target cells [36–39]. A recent study reported that psychosocial resources correlate with the expression of the cell surface maker CD57 (a marker of terminal maturation and senescence) on NK cells. These findings provide support that the sense that one has substantial resources may retard age-associated aspects of the microenvironment in which NK cells develop and mature, independent of effects on distress [40]. (2) Recurrence of MDD. The patients with single episode had significantly greater NKCA at baseline than those with recurrent episode. Recurrence of depression may contribute to immunologic decrease such as NKCA.

At present, major depressive disorders are diagnosed through patient interviews, symptom checklists based on diagnostic criteria of DSM-IV or DSM-5, self-reported scales, and scales by clinicians. However, there is a controversy over the objectivity of the assessment based on these symptoms and limitations on the establishment of an individualized treatment plan [41–43].

Biomarkers are indicators of normal biological processes, pathogenic processes, or pharmacological responses to a therapeutic intervention that can be measured and evaluated objectively [44]. However, the biomarker study of depression is difficult because of the heterogeneity of the disorder.

From the results, we carefully suggest that NKCA could be the possibility of being a diagnostic biomarker of anxiety-dominant depression subtype. CD+8 may also be an objective indicator of the severity and change of depression.

Results of this study in a limited small population could not be generalized to the patients with MDD. To overcome such a limitation, it is necessary to include normal control in the population and conduct in-depth, more systematic, wide-ranging research by assessing immune responses to antibodies and immune mediators, such as cytokine, as well as by counting immunocytes and by measuring their activity. An additional limitation to absent a healthy control sample is that it is difficult to determine whether immunological changes over time are related to technical changes in two assays or to experimental conditions unrelated to symptomatic changes in subjects.

It has been reported that a decrease of cell-mediated and innate immune responses is correlated with vulnerability to infectious diseases among patients with depression and that depression is correlated with immunologic activity among patients with cardiovascular diseases or inflammatory diseases, such as rheumatoid arthritis [5, 45–47]. These results imply that changes of immune functions among patients with MDD may depend on their age and chronicity of the condition, and such changes can also affect physical health of the patients.

Our research has provided new perspectives on the contribution of cellular immunity to major depression disorders and antidepressant treatment response as a potential biomarker.

## Conclusion

This study aimed to examine the immunologic changes by medication in patients with MDD, assess correlation between immunologic variables and symptom factors, and determine association between depression disorder and immune functions. Fifty-one patients with MDD who had no history of taking psychotropic drugs during the period of MDD episodes and had no major physical disease participated in this study. The 17-item Hamilton Rating Scale for Depression (HRSD) was used to assess the symptoms among the subjects, and the items in HRSD were divided into depression, insomnia, and anxiety factors by confirmational factor analysis. To assess the immune functions in the subjects, we used flow cytometry to count NK cells and lymphocytes in peripheral blood, employed ELISA using K-562 to assess activity of NK cells, and provided 4 week treatment with a selective serotonin inhibitor to observe changes of symptoms. We also counted NK cells and lymphocytes in peripheral blood, assessed their activity, and determined correlation between these variables and the three factors in HRSD. After treatment, the number of CD8-positive cells increased significantly (*p *< .05) and the CD4/CD8 ratio dropped significantly (*p *< .01). Positive correlation was found between the anxiety factors and NKCA at baseline (*r *= .36, *p *= .01); however, no correlation was found after treatment. As medication improved the symptoms of MDD, immune functions changed. The symptoms of anxiety, which accompanied MDD, were found to have a principal impact on such immunologic variables as NKCA.

## References

[1] Kessler RC, Berglund P, Demler O, Jin R, Koretz D, Merikangas KR, Rush AJ, Walters EE, Wang PS (2003). The epidemiology of major depressive disorder: results from the national comorbidity survey replication (NCS-R). JAMA.

[2] Penninx BW, Geerlings SW, Deeg DJ, van Eijk JT, van Tilburg W, Beekman AT (1999). Minor and major depression and the risk of death in older persons. Arch Gen Psychiatry.

[3] Rudisch B, NemeroV CB (2003). Epidemiology of comorbid coronary artery disease and depression. Biol Psychiatry.

[4] Evans DL, Ten Have TR, Douglas SD, Gettes DR, Morrison M, Chiappini MS, Brinker-Spence P, Job C, Mercer DE, Wang YL, Cruess D, Dube B, Dalen EA, Brown T, Bauer R, Petitto JM (2002). Association of depression with viral load, CD8 T lymphocytes, and natural killer cells in women with HIV infection. Am J Psychiatry.

[5] Zautra AJ, Yocum DC, Villanueva I, Smith B, Davis MC, Attrep J, Irwin M (2004). Immune activation and depression in women with rheumatoid arthritis. J Rheumatol.

[6] Irwin MR, Miller AH (2007). Depressive disorders and immunity: 20 years of progress and discovery. Brain Behav Immun.

[7] Sluzewska A (1999). Indicators of immune activation in depressed patients. Adv Exp Med Biol.

[8] Evans DL, Folds JD, Petitto JM, Golden RN, Pedersen CA, Corrigan M, Gilmore JH, Silva SG, Quade D, Ozer H (1992). Circulating natural killer cell phenotypes in men and women with major depression. Arch Gen Psychiatry.

[9] Herbert TB, Cohen S (1993). Depression and immunity—a meta-analytic review. Psychol Bull.

[10] Maes M, Lambrechts J, Bossman E, Jacobs J, Suy E, Vandervost C, DeJonekheere C, Mihner B, Raus J (1992). Evidence for a systemic immune activation during depression: results of leukocyte enumeration by flow cytometry in conjunction with monoclonal antibody staining. Psychol Med.

[11] Ravindran A, Griffith J, Merali Z, Anisman H (1999). Circulating lymphocyte subsets in obsessive compulsive disorder, major depression and normal controls. J Affect Disord.

[12] Farid Hosseini R, Jabbari Azad F, Talaee A, Miri S, Mokhber N, Farid Hosseini F, Esmaeili H, Mahmoudi M, Rafatpanah H, Mohammadi M (2007). Assessment of the immune system activity in Iranian patients with major depression disorder (MDD). Iran J Immunol.

[13] Zorrilla EP, Luborsky L, McKay JR, Rosenthal R, Houldin A, Tax A, Mccorkle R, Selgman DA, Schmidt K (2001). The relationship of depression and stressors to immunological assays: a meta- analytic review. Brain Behav Immun.

[14] American Psychiatric Association (2000). Diagnostic and statistical manual of mental disorders.

[15] Maes M, Stevens W, Peeters D, DeClerck L, Scharpe S, Bridts C, Schotte C, Cosyns P (1992). A study on the blunted natural killer cell activity in severely depressed patients. Life Sci.

[16] Grosse L, Carvalho LA, Birkenhager TK, Hoogendijk WJ, Kushner SA, Drexhage HA, Bergink V (2016). Circulating cytotoxic T cells and natural killer cells as potential predictors for antidepressant response in melancholic depression. Restoration of T regulatory cell populations after antidepressant therapy. Psychopharmacology (Berl).

[17] Hamilton M (1960). A rating scale for depression. J Neurol Neurosurg Psychiatry.

[18] O’Brien KP, Glaudin V (1988). Factorial structure and factor reliability of the Hamilton Rating Scale for Depression. Acta Psychiatr Scand.

[19] Hamilton M (1967). Development of a rating scale for primary depressive illness. Br J Soc Clin Psychol.

[20] Zheng YP, Zhao JP, Phillips M, Liu JB, Cai MF, Sun SQ, Huang MF (1988). Validity and reliability of the Chinese Hamilton Depression Rating Scale. Br J Psychiatry.

[21] Marcos T, Salamero M (1990). Factor study of the Hamilton Rating Scale for Depression and the Bech Melancholia Scale. Acta Psychiatr Scand.

[22] Fleck MP, Poirier-Littre MF, Guelfi JD, Bourdel MC, Loo H (1995). Factorial structure of the 17-item Hamilton Depression Rating Scale. Acta Psychiatr Scand.

[23] Bech P, Stage KB, Nair NP, Larsen JK, Kragh-Sørensen P, Gjerris A (1997). The Major Depression Rating Scale (MDS). Inter-rater reliability and validity across different settings in randomized moclobemide trials. Danish University Antidepressant Group. J Affect Disord.

[24] Freire-Garabal M, Nunez MJ, Losada C, Pereiro D, Riveiro MP, González-Patiño E, Mayán JM, Rey-Mendez M (1997). Effects of fluoxetine on the immunosuppressive response to stress in mice. Life Sci.

[25] Hernandez ME, Martinez-Fong D, Perez-Tapia M, Estrada-Garcia I, Estrada-Parra S, Pavón L (2010). Evaluation of the effect of selective serotonin-reuptake inhibitors on lymphocyte subsets in patients with a major depressive disorder. Eur Neuropsychopharmacol.

[26] Evans DL, Lynch KG, Benton T, Dubé B, Gettes DR, Tustin NB, Lai JP, Metzger D, Douglas SD (2008). Selective serotonin reuptake inhibitor and substance P antagonist enhancement of natural killer cell innate immunity in human immunodeficiency virus/acquired immunodeficiency syndrome. Biol Psychiatry.

[27] First MB, Spitzer RL, Gibbon M, Williams JBW (2000). Structured clinical interview for DSM-IV axis I disorders.

[28] Schleifer S, Keller S, Bartlett J (1999). Depression and immunity: clinical factors and therapeutic course. Psychiatry Res.

[29] Frank MG, Hendricks SE, Burke WJ, Johnson DR (2004). Clinical response augments NK cell activity independent of treatment modality: a randomized double-blind placebo controlled antidepressant trial. Psychol Med.

[30] Kessler RC, Nelson CB, McGonagle KA, Liu J, Swartz M, Blazer DG (1996). Comorbidity of DSM-III-R major depressive disorder in the general population: results from the US National Comorbidity Survey. Br J Psychiatry suppl.

[31] Fava M, Rankin MA, Wright EC, Alpert JE, Nierenberg AA, Pava J, Rosenbaum JF (2000). Anxiety disorders in major depression. Compr Psychiatry.

[32] Philip TN, Joseph B (2001). Symptomatic and syndromal anxiety and depression. Depress Anxiety.

[33] Nemeroff CB (2002). Comorbidity of mood and anxiety disorders: the rule, not the exception?. Am J Psychiatry.

[34] Andreoli A, Keller SE, Rabaeus M, Zaugg L, Garrone G, Taban C (1992). Immunity, major depression, and panic disorder comorbidity. Biol Psychiatry.

[35] Tashiro M, Itoh M, Kubota K, Kumano H, Masud MM, Moser E, Arai H, Sasaki H (2001). Relationship between trait anxiety and natural killer cell activity in cancer patients: a preliminary PET study. Psychooncology.

[36] Fiatarone MA, Morley JE, Bloom ET, Benton D, Solomon GF, Makinodan T (1989). The effect of exercise on natural killer cell activity in young and old subjects. J Gerontol.

[37] Grubeck-Loebenstein B, Della Bella S, Iorio AM, Michel JP, Pawelec G, Solana R (2009). Immunosenescence and vaccine failure in the elderly. Aging Clin Exp Res.

[38] Miyaji C, Watanabe H, Minagawa M, Toma H, Kawamura T, Nohara Y, Nozaki H, Sato Y, Abo T (1997). Numerical and functional characteristics of lymphocyte subsets in centenarians. J Clin Immunol.

[39] Rukavina D, Laskarin G, Rubesa G, Strbo N, Bedenicki I, Manestar D, Glavas M, Christmas SE, Podack ER (1998). Age-related decline of perforin expression in human cytotoxic T lymphocytes and natural killer cells. Blood.

[40] Segerstrom SC, Al-Attar A, Lutz CT (2012). Psychosocial resources, aging, and natural killer cell terminal maturity. Psychol Aging.

[41] Hilsenroth MJ, Baity MW, Mooney MA, Meyer GJ (2004). DSM-IV major depressive episode criteria: an evaluation of reliability and validity across three different ratingmethods. Int J Psychiatr Clin Pract.

[42] Phillips J, Frances A, Cerullo MA, Chardavoyne J, Decker HS, First MB, Ghaemi N, Greenberg G, Hinderliter AC, Kinghorn WA, LoBello SG, Martin EB, Mishara AL, Paris J, Pierre JM, Pies RW, Pincus HA, Porter D, Pouncey C, Schwartz MA, Szasz T, Wakefield JC, Waterman GS, Whooley O, Zachar P (2012). The six most essential questions in psychiatric diagnosis: a pluralogue part 1: conceptual and definitional issues in psychiatric diagnosis. Philos Ethics Humanit Med.

[43] Stein DJ, Phillips KA, Bolton D, Fulford KW, Sadler JZ, Kendler KS (2010). What is a mental/psychiatric disorder? From DSM-IV to DSM-V. Psychol Med.

[44] Biomarkers Definitions Working Group (2001). Biomarkers and surrogate endpoints: preferred definitions and conceptual framework. Clin Pharmacol Ther.

[45] Cohen S, Miller G, Ader R, Felten D, Cohen N (2000). Stress, immunity and susceptibility to upper respiratory infection. Psychoneuroimmunology.

[46] Lesperance F, Frasure-Smith N, Theroux P, Irwin M (2004). The association between major depression and levels of soluble intercellular adhesion molecule 1, interleukin-6, and C-reactive protein in patients with recent acute coronary syndromes. Am J Psychiatry.

[47] Miller GE, Stetler CA, Carney RM, Freedland KE, Banks WA (2002). Clinical depression and inflammatory risk markers for coronary heart disease. Am J Cardiol.

